# Contextual Intervention Adapted for Autism Spectrum Disorder: An RCT of a Parenting Program with Parents of Children Diagnosed with Autism Spectrum Disorder (ASD)

**Published:** 2019

**Authors:** Zahra PASHAZADEH AZARI, Seyed Ali HOSSEINI, Mehdi RASSAFIANI, Sayyed Ali SAMADI, Samaneh HOSEINZADEH, Winnie DUNN

**Affiliations:** 1 Department of Occupational Therapy,University of Social Welfare and Rehabilitation Sciences, Tehran, Iran; 2 Department of Occupational Therapy, School of Rehabilitation, Shahid Beheshti University of Medical Sciences, Tehran, Iran; 3 Pediatric Neurorehabilitation Research Center, University of Social Welfare and Rehabilitation Sciences, Tehran, Iran; 4 Department of Life and Health Science, University of Ulster, London, UK; 5 Biostatistics Department, University of Social Welfare & RehabilitationSciences, Tehran, Iran; 6 Department of Occupational Therapy, University of Missouri, Columbia, USA

**Keywords:** Contextual intervention, Sensory processing patterns, Coaching, Autism spectrum disorders

## Abstract

**Objectives:**

We investigated the effects of a manualized Contextual Intervention adapted for Autism Spectrum Disorders (CI-ASD), and essential elements of the intervention in promoting children’s participation and mothers’ parenting self-efficacy.

**Materials & Methods:**

In this randomized controlled trial, conducted in Tehran, Iran in 2017, participants (36 parents of children with ASD) were randomly assigned to wait-list control or intervention groups. The intervention comprised contextually reﬂective occupational therapy combines 3 elements: sensory processing patterns, coaching, and social support. We provided the program to promote child’s participation and parent’s efficiency. During phase 1, the participants in the intervention group received CI-ASD as long as Treatment As Usual (TAU) and during phase 2 they received TAU only. We completed the outcome measures at three-time points (pre-intervention, post-intervention, and follow-up). We conducted semi-structured interviews post-intervention to explore acceptability of intervention and participants’ experiences of CI-ASD.

**Results:**

CI-ASD can produce meaningful effects in eliminating sensory issues, promoting child participation and parenting efficiency in ASD families, compared to TAU. Parents reported high levels of acceptance and also confirmed the family’s achievements.

**Conclusion:**

These gains suggest CI-ASD as an effective intervention for children who have ASD and their families, but further studies are needed to declare and generalize the findings over time. Estimated effect sizes were in the large and medium ranges and favored the intervention group.

## Introduction

Enabling participation in everyday occupations for children with disabilities has become an important outcome for rehabilitation services (1). Participation in life activities is a critical factor in children’s development and facilitates learning (2). Participation is defined as the nature and extent of a person’s involvement in life situations, denoting the interplay of the person, environment, and activity (3). As occupational therapists, we have unique skills to act within this interaction and understand the impact of the occupations and the environment on participation. We also see the possibilities for adapting occupations and environments to optimize the child’s functioning in natural contexts (4). 

Children with autism spectrum disorder (ASD) may demonstrate unusual responses to sensory stimuli and may demonstrate bizarre interests in sensory features of the contexts (5). This can influence their participation in daily activities (6). A disparity between environmental demands and child’s sensory processing patterns can contribute to less participation (7). Occupational therapists may embed sensory inputs within a child’s daily routines to modulate arousal level or adapt home or school environments to promote participation (8).

Occupational performance coaching (OPC), or simply “coaching” is an intervention has recently begun to receive attention in the early intervention literature and is practiced in family- centered programs which supports parent-identified goals and problem solving. Coaching enables parents to realize and carry out therapeutic strategies within life routines (9, 10). The coach does not “tell” parents what to do, instead guides them in identifying therapeutic strategies according to families’ needs (11, 12). Coaching has a conversational format that guides parents to identify their functional goals and determine adjustments in activities and natural environments that promote goal achievement within routines and authentic contexts. The coach may also use shaping and processing strategies to improve parent’s recognition and problem-solving (13).

Although the literature inform therapists on how to administer effective coaching services (13, 14), limited clues exist about using sensory processing knowledge combined with a coaching approach. 

**Figure 1:**
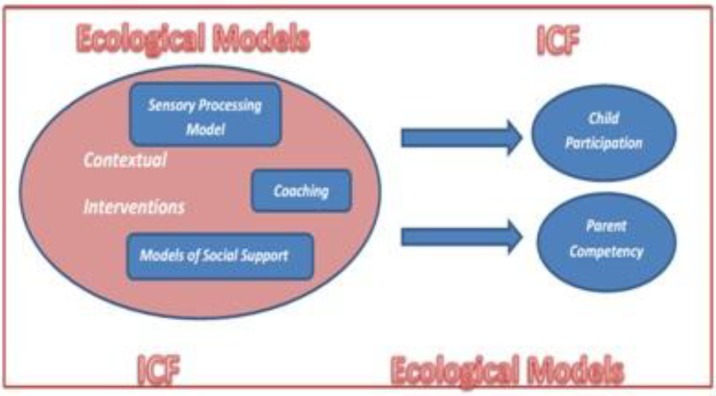
Illustration of CI-ASD and outcomes Modified illustration from “Impact of a Contextual Intervention on Child Participation and Parent Competence among Children with Autism Spectrum Disorders: A Pretest-Posttest Repeated-Measures Design” by W. Dunn et al. 2012, American Journal of Occupational Therapy, 66(5):520-8

We hypothesized that implementing a contextual intervention adapted for Autism Spectrum Disorder (CI-ASD) within family activities enlightened by child’s sensory processing patterns could improve occupational performance and parental self-efficacy (Figure 1). Using Dunn’s Sensory Processing Framework (2014) we used a contextual intervention to examine the efficacy of CI-ASD, and its acceptability among parents. Our questions were as follows:

1. Does CI-ASD promote children’s participation in family activities and routines?

2. Does CI-ASD promote parenting sense of efficacy?

3. What is the intervention acceptability and participation rate?

## Materials & Methods

**Research Design**

In the current research we used a randomized controlled trial with a mixed within-between-subjects design and a wait-list control group. We completed randomization by writing children’s names at random and allocating to the intervention and wait-list control groups, using a randomization block. No parties were blinded to group allocation.

Before starting the intervention, we completed the pre-intervention assessments with both the intervention and the wait-list control groups. The intervention group then received the CI-ASD and at the end of the intervention course, both groups completed the same post-intervention measures. We also conducted semi-structured interviews to investigate parents’ experience (satisfaction) of CI-ASD. Four weeks later, we conducted another round of assessments with both groups (follow up).

The wait-list control group received CI-ASD after follow-up and treatments as usual (TAU) continued for both groups, all the study long. We recorded other treatment services received by participants but did not control for them.

We obtained ethical clearance for the research from the Ethics Committee at the University of Social Welfare and Rehabilitation Sciences (IR.USWR.REC.1395.189).

**Participants**

The participants were parents of children ages 3-10 (at point of recruitment) with ASD (based on prior diagnosis). The participating parents reported their child’s diagnosis as ASD. Before starting the study informed consent was obtained from all participating parents. Recruitment was based upon parent report, SSP data, demographic questionnaire, and informed consent. All children included in this study had at least one sensory pattern outside typical range based on the Short Sensory Profile II. 

Ethical clearance was obtained by the ethics committee at the University of Social Welfare and Rehabilitation Sciences (IR.USWR.REC.1395.189).

**Fig.2:**
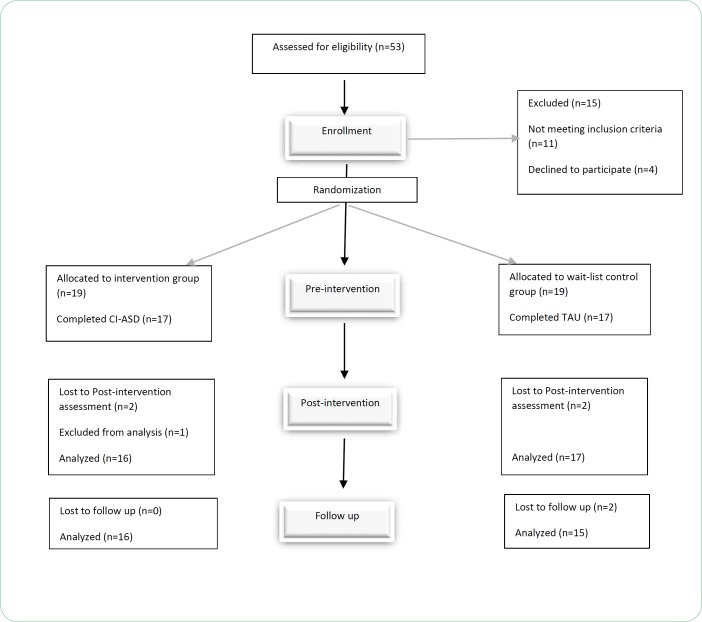
CONSORT flowchart

We recruited thirty-eight families from two rehabilitation centers (Navid-e-asr and Omid-e-asr rehabilitation centers in Tehran, Iran in Summer 2017), 19 were randomized into the intervention group and 19 were randomized into the wait-list control group. Mostly the mothers completed the program and the questionnaires. Mothers and If available, fathers attended intervention sessions. The flowchart of the study is illustrated in Figure 2.

**Measures**

The Demographic Questionnaire, Short Sensory Profile II (SSP2), and Gilliam Autism Rating Scale II (GARS2) were only completed in the pre-intervention questionnaire pack. Parents completed all other questionnaire packs before and after the intervention, and at 4 wk follow-up. 

***Demographic Questionnaire***

The Demographic Questionnaire contains the family background information, the child’s data, received services, and contact details.

***Sensory Profile II***

We used the Short Sensory Profile II (SSP2), a 38-item parent questionnaire, to identify children who have sensory differences. According to its short administration time (5-10 min) and value in screening for sensory processing patterns, the SSP is recommended for research protocols (15, 16). The questionnaire evaluates behaviors associated with sensory processing in children aged 3–10 yr (17). Based on a 5 point Likert scale ranging from ‘always’ [1] to ‘never’ [5], parents rate the frequency of various sensory behaviors of their child. 

The SSP contains seven sections: taste/smell, tactile, movement, low energy/weak, auditory filtering, visual/auditory sensitivity, under-responsive/seeks sensation and general functions of sensory modulation. Low scores show frequent sensory behaviors. The SSP provides normative data that categorize a child’s score in each section in ‘typical performance’, ‘probable difference’ or ‘definite difference’. Psychometric properties include internal consistency ranging from 0.70 to 0.90 (18, 19) and discriminative validity more than 95% in distinction of atypical sensory processing patterns. The total Cronbach’s alpha coefficients were reported 0.874, implies good internal reliability of the questionnaire (20). Inter-scale correlations were reported from 0.25 to 0.76, suggesting that the subscales measure unique dimensions. The total score is the most sensitive indicator of sensory patterns (19, 21). The Persian version of SSP was carried out for Iranian children 5 to 12 yr of age in 2011 in which the validity and reliability were above 90% ((22). 

***Gilliam Autism Rating Scale II***

The second version of Gilliam Autism Rating Scale (GARS2) is a behavior questionnaire that comprise 42 items classified into three parts: Stereotyped Behaviors, Communication and Social Interaction, for persons aged 3–22 year. Based on 4 degrees ranging from Never Observed (0) to Frequently Observed (3) caregivers rated child’s present behavior. Regarding American normative data of children with ASD, the scores in each part are totaled and derived a standard score. 

The scores imply the likelihood of having an ASD which grouped in one of three conditions ‘‘unlikely, possibly and very likely’’. Additional 14 items were added in the revised edition of GARS that afford information about the development of first three years of child’s life (23). According to normative data of 1107 individuals with ASD and 328 non-ASD persons and those with other developmental disabilities, validity and reliability data for the English version of GARS is on hand. Test-retest and internal consistency for the Autism Index and for the subscales range from 0.80 to 0.90 (24). 

The Persian version of GARS II was completed by parents of 658 children: 442 with autism; 112 intellectually disabled and 102 normally developing. Using Chronbach’s Alpha coefficients, the internal consistency for subscales and total items were calculated on GARS for Persian manuscript declared acceptably high (from 0.84 to 0.95). Test-retest reliability were calculated for the three subscales and total score which were highly significant (0.959 to 1.000). Discriminative validity, across the three subgroups of children (Autism, intellectual disability, normal development) were identified for total scores and sub-scales on the Persian version of GARS (*P*˂0.001) (25).

The Persian version of GARS II was examined for language clarity and appropriateness for use in Iranian culture. Five of the 42 questions were unclear to parents and these items were reworded for greater clarity (26).

***Canadian Occupational Performance Measure***

We used the Canadian Occupational Performance Measure [COPM; (27)] to identify problems concerning children’s daily life (self-care, productivity, and leisure) and parents are asked to identify the problems associated with sensory responses. The importance of each problem is graded on a scale ranging from 1 to 10. The parents selected five problems that had greater importance and graded their satisfaction and child’s performance on a scale ranging from 1 to 10. Lower grading denotes less satisfaction and worse performance. The parent-identified problems made our intervention goals and raised scores imply met outcomes. Psychometric properties comprise test-retest reliability of 0.80 for performance and 0.89 for satisfaction, and internal consistencies of 0.56 and 0.71 for performance and satisfaction scores (27, 28). 

***Goal Attainment Scaling***

We used Goal Attainment Scaling [GAS; (29)]to measure improvement in functional goals in activities and routines related sensory responses. The inter-rater reliability of the scale was declared 0.67 in various populations (30). In our study, parent and intervention therapist found prevailing problems related to sensory issues and made incremental levels into goal achievement. Each goal was rated on a 5-point scale (-2, -1, 0, +1, +2) and the current behavior was set at the level of (-2) and ultimately parents checked the level of each goal progress. If the parent obtains the expected level of identified-goal, it was graded at 0. If they obtain less than expected level it was graded at -1 and -2; if they obtain more than the expected level it was scored at +1 and +2. Evidence have suggested the GAS for measuring parents’ statements of behavioral variations (12, 31).

***Parenting Sense of Efficacy Measure***

The Parenting Sense of Efficacy Measure (PSEM) is a 10-item questionnaire (responses range from 1=strongly disagree to 7= strongly agree) that measures parental self-efficacy and sense of competency. The scoring items of 1,3,5,6, and 8 is in reverse mode. Upper scores denote more sense of competency and more efficacy. The Persian version of PSEM was carried out for Iranian parents in 2011 in which the validity declared acceptably high and using Chronbach’s Alpha coefficients, the internal consistency was calculated above 80% (32).

**Intervention Procedure**

The first author (the intervention therapist, coach), an occupational therapist, with 20 yr of clinical experience in pediatric rehabilitation implemented CI-ASD sessions during the study. The coach provided 2 training group sessions and 10 individual sessions of coaching (over 11 wk) for each mother to recognize strategies for improving their child’s participation to achieve functional goals. Mostly the target child did not attend training group and coaching sessions; during the intervention period, children continued to receive treatments as usual (TAU). The target fathers and children were welcome to attend intervention sessions if available.

During training group sessions, the coach established rapport with mothers and shared basic information about sensory processing patterns based on Dunn’s sensory processing model. The coach talked about how child’s behavioral responses and daily routines might be affected, ways to adapt the child’s environment/ activities and how to improve self-regulation strategies. The coach also provided a booklet with pictures to promote sharing knowledge and make information meaningful, relevant and integrated to mothers.

Coaching sessions began with reviewing the parent performance goals using COPM and GAS and activity configuration (i.e., outline of family’s routines). The coach and parent designed intervention plans for the next week, identifying child’s sensory processing patterns, using SSP data. The plans mirrored how sensory processing knowledge enlightened the family programs in activities and routines. During the sessions, the coach and parent collaboratively analyzed the identified functional goals, using the sensory processing model. They discussed what had happened since the last session and the coach continued to provide guidance through the problem-solving process. At the end of each session, coach and parent created a shared design clarifying the family’s programs.

At the end of intervention period, we investigated parents’ experience of CI-ASD, using a semi-structured interview.

***Intervention Protocol***

The adaptation of the Contextual Interventions for ASD (CI-ASD) and the theoretical underpinning are described in detail elsewhere (Pashazadeh, Hosseini, Rassafiani, Dunn & Samadi, in press). Parents attend 2 training group sessions and 10 weekly, 45 min individual sessions of coaching. The intervention contains three treatment characteristics: 1) sensory processing knowledge, 2) coaching and 3) social support. 

During the training group sessions, therapist associates sensory processing principles to the child’s activities and daily life, in this way parents determine child’s sensory processing patterns and its effect on performance. Considering the effect of each child’s sensory processing patterns on participation, Short Sensory Profile II (SSP2) facts can inform the coach and parent about which aspects of tasks might be easy or challenging for the child.

Consistent with Occupational Performance Coaching, CI-ASD arose from three empowering components of coaching: adult learning, setting goals, and a strength-based approach (12, 13). The CI-ASD process involves developing a therapeutic coaching relationship, which provides the milieu for learning about sensory processing challenges of their child, supports selection of identified functional goals associated with sensory behaviors, and enables the parent to take the steps through the problem-solving process. 

The therapist/coach support to parents’ performance in executing plans is an important feature of this process. The coach guides parents’ recognition and planning strategies for making a better match between child, environment, and activity components to promote participation in everyday life. In the subsequent sessions, the coaching therapist notices the influence of the strategies on child’s participation in partnership with the parent. Therapist asks reflective questions throughout sessions to cue parents to deliberate about what other places, times, or situations that they might also promote function in daily life. Mostly sessions terminate with writing parents’ strategies and explanation of planned actions for the coming week. The coaching relationship provides emotional support that creates a safe environment of trust, and mutual relationship for sharing information, identifying options, and progressing toward identified goals (33). 

**Statistical Analysis**

A series of *t*-test was performed for comparing means of responses in two groups. Mauchly test was performed to sphericity assumption in repeated measures ANOVA. Due to the assumption was not established (*P*-value<0.05), the test with adjusted degree of freedom was used (Greenhouse-Geisser). 

We applied a series of repeated measures ANOVAs to explore intervention effects and maintenance. These analyses used data from the two groups and compared pre-intervention assessments data to data at post-intervention and follow-up. The reported results are group effects, time effects and the interaction effects (2 groups x 3 times interaction). Due to the results of the group effects and interaction effects (group x time interaction), Bonferroni adjustment was applied to compare responses in each group within measuring steps, also for comparing two groups, in each measuring steps, MANOVA coefficients estimated. We used SPSS ver. 21 (Chicago, IL, USA) and selected the Level of significant level at 0.05.

## Results

**Participants Characteristics**

The power analysis indicated that for a large effect it was necessary to enroll 17 participants per group. Participants were parents of 38 children ages 3-10 yr (Mean intervention group=6.5 yr; Mean control group=7.12 yr) who had a diagnosis of ASD. Parents reported household income levels (Low: 36%; Medium: 64%). Majority of the participating parents had diploma or under diploma (79%) and minority of them had some college education (21%). Mostly fathers did not follow therapeutic sessions, so the main of the participating parents were mothers (94%). All children received other services (ABA, speech therapy, group therapy, medication) throughout the study.

There were no meaningful differences at pre-intervention assessments between two groups in the most participant’s characteristics, using Chi-Square, *t*-test and Fisher exact test as appropriate (Table 1).

**Table 1:** Participants’ data for two groups at baseline

Variables	Intervention (n=16)	Control (n=17)	Combined (N=33)	T value/ X^2 ^value	*P*-value
Age of target child	6.50 ± 2.098	7.12 ± 2.643	6.82 ± 2.378	-.746	.462
Sex of the child					
Male Female	14 2	12 5	26 (79%) 7 (21%)	1.411	.235
Sensory Processing issues of the child (SSP data)					
3-4 patterns 5-6 patterns 7 patterns	4 6 6	3 8 6	7 (21%) 14 (42.5%) 12 (36.5%)	5.203	.267
Level of function (GARS data)					
Low/ Moderate function High function	10 6	11 6	21 (64%) 12 (36%)	.017	.895
Number of children in the family					
One Two Three/four	9 4 3	6 6 5	15 (45.5%) 10 (30.5%) 8 (24%)	1.471	.479
Participating parent					
Mother Father	16 0	15 2	31 (94%) 2 (6%)	-	.485*
Age of participating parent					
20-30 31-40 41-55	5 9 2	2 9 6	7 (21%) 18 (55%) 8 (24%)	3.258	.196
Employment status of the participating parent					
Employed Not employed	2 14	2 15	4 (12%) 29 (88%)	-	1.000*
Education of participating parent					
Low education Some college degree	15 1	11 6	26 (79%) 7 (21%)	4.160	.041
Family income					
Low Medium	3 13	9 8	12 (36%) 21 (64%)	4.164	.041

*Fisher Exact Test is used.

**Table 2:** Outcome variables for two groups at baseline

Outcome variables	Group	Mean	Std. Deviation	P-value
COPM Performance	Int	12.38	4.031	.511
	Con	11.18	6.054	
COPM Satisfaction	Int	11.50	3.916	.823
	Con	11.94	6.977	
GAS	Int	-6.00	.000	-
	Con	-6.00	.000	
PSEM	Int	45.00	7.806	.528
	Con	43.06	9.510	

**Table 3:** The abstract of Repeated Measures ANOVA results

Outcome Measures	Time		group		Time x group interaction	
	*P*-value	Effect size	*P*-value	Effect size	*P*-value	Effect size
COPM Performance	˂ .001**	.776	˂ .001**	.229	˂ .001**	.397
COPM Satisfaction	˂ .001**	.784	.007*	.229	˂ .001**	.457
GAS	˂ .001**	.853	˂ .001**	.694	˂ .001**	.598
PSEM	.050	.100	.059	.118	.013*	.144

**Table 4:** The means and effect size for the outcome measures in two groups

Outcome Measures	Pre-intervention		Post-intervention		Follow-up		Effect size	
	Int	Con	Int	Con	Int	Con	Int	Con
COPM Performance	12.38±4.031	11.18±6.054	25.94±7.047	16.94±9.698	29.44± 7.136	17.73± 7.887	87%	82%
COPM Satisfaction	11.50±3.916	11.94±6.977	27.94±6.904	17.35 ± 10.142	30.75± 6.547	18.67± 8.550	87%	54%
GAS	-6.00± 0.000	-6.00± .000	1.75 ±2.266	-3.88 ± .965	2.88± 2.217	-2.93± 1.831	92%	63%
PSEM	45.00±0.806	43.06±9.510	51.25±7.206	42.06 ± 9.871	51.13± 9.040	42.93± 12.389	39%	.6%

**Table 5:** The abstract of Bonferroni test results

Comparing Steps	Group	COPM performance		COPM satisfaction		GAS		PSEM	
		*P*-value	Means Difference	*P*-value	Means Difference	*P*-value	Means Difference	*P*-value	Means Difference
Pre- Post	Int	˂ .001	13.563**	˂ .001	16.438**	˂ .001	7.750**	.006	6.250*
	Con	.01	6.200*	.003	6.000*	.001	2.333*	1.000	.800
Post- Follow	Int	˂ .001	3,500**	.002	2.813*	.063	1.125	1.000	.125
	Con	1.000	1.33	1.000	.600	.230	.733	1.000	.333

**Table 6:** The abstract of MANOVA coefficients ANOVAs to explore intervention effects and maintenance. These analyses used datafrom the two groups and compared pre-intervention assessments data to data at post-intervention and follow-up

Measurement	Group	COPM performance		COPM satisfaction		GAS		PSEM	
		P-value	B	*P*-value	B	*P*-value	B	*P*-value	B
Pre	Int	.606	.975	.783	-.567	-	1.003	.624	1.600
	Con	-	-	-	-	-	-	-	-
Post	Int	.011	8.338	.004	9.871	˂ .001	5.417	.011	8.650
	Con	-	-	-	-	-	-	-	-
Follow	Int	˂.001	11.704	˂ .001	12.083	˂ .001	5.808	.043	8.192
	Con	-	-	-		-	-	-	-

We did not find any notable differences within the intervention and the wait list groups independent variables at baseline, using Independent *t*-test (Table 2). The preliminary analyses corroborate the assumption of between-group comparability at the start.

Attrition was 13% (5/38) overall, 16% (3/19) in CI-ASD, and 11% (2/19) in TAU group. Due to the mother’s problematic pregnancy (one participant), home transfer (one participant), and no tendency to continue the program (one participant), attrition occurred in the intervention group. Thus, 16 of the 19 children enrolled in CI-ASD completed the intervention. Attrition in the control group occurred due to the home transfer (two participants). Thus 17 of the 19 enrolled in TAU completed intervention (Figure 2). 

**Primary Outcomes**

As stated before, our research hypothesis was that, the participants in the intervention group indicate greater gains in children’s participation and parenting efficacy, relative to the control group. Repeated measures ANOVA showed that the time, group and time x group interaction had significant effects on COPM performance (*P* ˂ .001), COPM satisfaction (*P* ˂ .001), GAS scores (*P* ˂ .001) and PSEM scores (*P* ˂ .013). Only group effects were not significant for PSEM (*P* = .059).

Due to the results of the group effects and interaction effects (group x time interaction), we employed a Bonferroni adjustment to compare responses within and across groups. Table 4 provides the means and effect sizes for all of the primary outcome measures. In line with the hypotheses, the estimated effect sizes preferred participants in the intervention group.

The results of ANOVA for COPM performance yielded a notable time x group increase for performance, F=9.093, *P*<0.001, η2=0.397; and a significant time effect reporting increasing children’s performance in the steps of assessments, F=100.522, *P*<0.001, η2=0.776; and a significant group effect reporting difference between two groups, F= 8.625, *P*<0.001, η2=0.229. Time effect in the intervention group was 87% and in the control group 82%.

The results of ANOVA for COPM satisfaction exhibited a notable time x group change for satisfaction of performance, F= 24.394, *P*<0.001, η2=0.457; and a significant time effect reporting increasing satisfaction of performance in the steps of assessments, F=105.401, *P*<0.001, η2=0.784; and a significant group effect reporting difference between two groups, F=8.591, p<0.007, η2=0.229. Time effect in the intervention group was 87% and in the control group 54%.

The results of ANOVA for GAS revealed a significant time x group effect for goal attainment, F=43.058, *P*<0.001, η2=0.598; and a significant time effect reporting increasing goal attainment scores within measuring steps, F=168.697, *P*<0.001, η2=0.853; and a significant group effect reporting increasing goal attainment scores between two groups, F=65.888, *P*<0.001, η2=0.694. Time effect in the intervention group was 92% and in the control group 63%.

The results of ANOVA for PSEM revealed a significant time x group effect for parental self-efficacy, F=4.877, *P*<0.013, η2=0.144; and a significant time effect reporting increasing in parenting self-efficacy scores within measuring steps, F=3.226, *P*<0.050, η2=0.100; and a significant group effect reporting difference between two groups , F=3.868, *P*<0.059, η2=0.118. Time effect in the intervention group was 39% and in the wait-list control group .6%.

Due to the results of the group effects and interaction effects (2 groups x 3 times), Bonferroni adjustment was applied to compare responses in each group within measuring steps.

Bonferroni tests indicated significant differences between means of COPM performance in pre-post, 13.563 (*P*˂0.001), and post-follow, 3.500 (*P*˂0.001) in the intervention group, and differences between means in pre-post, 6.200 (*P*=0.01) and post-follow, 0.133 (*P*=1.000) in control group.

Bonferroni tests indicated significant differences between means of COPM satisfaction in pre-post, 16.438 (*P*˂0.001), and post-follow, 2.813 (*P*=0.002) in the intervention group, and differences between means in pre-post, 6.000 (*P*=0.003) and post-follow, 0.600 (*P*=1.000) in control group.

Bonferroni tests indicated significant difference between means of GAS scores in pre-post, 7.750 (*P*˂.001), and maintained results to follow-up (*P*=0.063) in the intervention group. Significant difference between means in pre-post, 2.333 (*P*=0.001) was detected and maintained results to follow-up (*P*=0.230) in control group.

Bonferroni tests indicated significant differences between means of PSEM in pre-post, 6.250 (*P*=0.006), and maintained results to follow-up in the intervention group. No significant differences between means in pre-post, 0.800 (*P*=1.000) and post-follow, 0.333 (*P*=1.000) was detected in control group (Table 5).

A series of MANOVA coefficients estimated for comparing two groups, in each measuring steps (Table 6).

The MANOVA coefficients contrast between two groups for COPM performance indicated signiﬁcant differences for Performance scores in the post-intervention (t=2.712, *P*=0.011) and follow-up (t=4.337, *P*<0.001) and no significant difference in pre-intervention (t=0.522, *P*=0.606). 

MANOVA coefficients contrast between two groups for COPM satisfaction showed notable differences for satisfaction points in post- intervention (t=3.136, *P*=0.004) and follow-up (t=4.435, *P*<0.001) and no significant effect in pre-intervention (t= -0.278, *P*=0.783).

MANOVA coefficients contrast between two groups for GAS evidenced a meaningful increase on goal attainment points in post-intervention measures (t=7.055, *P*˂0.001) and follow-up (t=7.922, *P*< .001) and revealed no significant difference in pre-intervention (t = -, *P*= -).

MANOVA coefficients contrast between two groups for PSEM indicated a signiﬁcant effect for Parenting Self-efficacy scores in the post- intervention (t=2.707, *P*=0.011) and follow-up (t=2.113, *P*=0.043) and no significant difference in pre-intervention (t=0.495, *P*=0.634). 

**Secondary Outcomes**

Our research question was about the intervention acceptability and participation rate. Across the intervention group, the majority of parents rated the intervention acceptability high (according to the treatment acceptability questionnaire), with a mean of 30.88 (SD=1.258), which scores range from 1 to 32. Parents in intervention group had high adherence as rated (the scores range from 1 to 10) by the coach (Mean=8.63, SD=1.258), with higher scores indicating greater adherence. We employed descriptive statistics to evaluate participation rate, that is, the percentage of participants who allocated and completed the study. The intervention group had an 89% completion rate (17/19), and ten percent of participants dropped out and did not complete the program (2/19). 

## Discussion

In both groups findings reveal improved participation in children; however, in line with our hypothesis, we found signiﬁcant difference between two groups, in favor of the intervention group. There were clinical meaningful increases of child participation within the intervention group when comparing to the control group on the COPM performance, COPM satisfaction, and the GAS. Additionally, analysis of the follow-up data showed that increases continued in the intervention group, but not in the control group. 

A significant improvement in parenting efficacy was evident in the intervention group in comparison to the wait-list group. At the end of CI-ASD parents in the intervention group showed significant increase of self-efficiency assessed by the Parenting Self-efficacy Measure. The changes were maintained 4 wks. after the intervention period in the intervention group. Hence, CI-ASD is an efficacious program on child’s sensory behavior issues and parenting dilemmas in the ASD families, in line with our hypothesis.

The participation rate of the intervention group was high, and the majority of participating parents completed the course of the intervention. Participants in intervention group showed high adherence as rated by the coach, which higher scores indicating greater adherence. Among the intervention group, parents reported high levels of acceptance. Higher scores showed that the procedure and outcomes of CI-ASD were favorably viewed by parents, and having little difficulty implementing the intervention strategies in life routines.

The gains of the current study are consistent with the finding of previous studies showing the effects of parent empowerment (34, 35). Parallel to our finding, preceding studies have shown signiﬁcant participation gains in children and promotion in parents’ sense of competence in parenting after implementing occupational performance coaching (13, 36). 

Even though these two published types of research declared that their intervention was effective in promoting participation and performance of children, we did not have a control group. The current study explored the intervention effectiveness in an RCT that included a control group and consistent with Contextual Intervention, we used sensory processing model integrated with coaching approach. All of the primary outcome measures, were statistically significant and three of them evolved potentially high effect size estimates.

It is required to run further researches to evolve evidence for CI-ASD and establish an apparent guideline of the intervention so we can repeat it and gain the same results with other practitioners. AS this small sample acquire good power and effect size estimates, revealed that this program has assurance. Describing the most impressive components of the intervention procedure can help in training experienced occupational therapists and shifting into practice.

**Limitations**

We did not have blinded assessment in the present study and we had a short length of time to follow-up. After the study, additional researches need to be trained so that others can learn and carry out CI-ASD with desired results and fidelity. Future studies could include observational assessments of participation and self-efficacy to expand the data. 

**In conclusion, **the gains of the present study reveal that the CI-ASD program is efficacious in eliminating children’s sensory behavior issues and promoting participation and performance reported by parents and the findings provide support for the efficacy of the program in parent outcomes in the ASD families. This program has assurance for the larger community and needs additional researches.
